# Assessing the vertical transmission potential of dengue virus in field-reared *Aedes aegypti* using patient-derived blood meals in Ho Chi Minh City, Vietnam

**DOI:** 10.1186/s13071-020-04334-5

**Published:** 2020-09-14

**Authors:** Daniela da Silva Goncalves, Kien Duong Thi Hue, Vi Tran Thuy, Nhu Vu Tuyet, Giang Nguyen Thi, Van Huynh Thi Thuy, Trang Huynh Thi Xuan, Dui Le Thi, Long Thi Vo, Huynh Le Anh Huy, Nguyen Thi Van Thuy, Bridget A. Wills, Phong Nguyen Thanh, Cameron P. Simmons, Lauren B. Carrington

**Affiliations:** 1 Oxford University Clinical Research Unit, Wellcome Trust Major Overseas Programme, District 5, Ho Chi Minh City, Vietnam; 2 Hospital for Tropical Diseases, District 5, Ho Chi Minh City, Vietnam; 3 Institute for Vector Borne Disease, Monash University, Clayton, Melbourne, VIC 3168 Australia

**Keywords:** Vertical transmission, Dengue virus (DENV), Mosquitoes, *Aedes aegypti*, *Aedes albopictus*

## Abstract

**Background:**

Dengue viruses (DENV) can be transmitted from an adult female *Aedes aegypti* mosquito through the germ line to the progeny; however, there is uncertainty if this occurs at a frequency that is epidemiologically significant. We measured vertical transmission of DENV from field-reared *Ae. aegypti* to their F1 progeny after feeding upon blood from dengue patients. We also examined the transmission potential of F1 females.

**Methods:**

We examined the frequency of vertical transmission in field-reared mosquitoes, who fed upon blood from acutely viremic dengue patients, and the capacity for vertically infected females to subsequently transmit virus horizontally, in two sets of experiments: (i) compared vertical transmission frequency of field-reared *Ae. aegypti* and *Ae. albopictus*, in individual progeny; and (ii) in pooled progeny derived from field- and laboratory-reared *Ae. aegypti.*

**Results:**

Of 41 DENV-infected and isofemaled females who laid eggs, only a single female (2.43%) transmitted virus to one of the F1 progeny, but this F1 female did not have detectable virus in the saliva when 14 days-old. We complemented this initial study by testing for vertical transmission in another 460 field-reared females and > 900 laboratory-reared counterparts but failed to provide any further evidence of vertical virus transmission.

**Conclusions:**

In summary, these results using field-reared mosquitoes and viremic blood from dengue cases suggest that vertical transmission is uncommon. Field-based studies that build on these observations are needed to better define the contribution of vertical DENV transmission to dengue epidemiology.
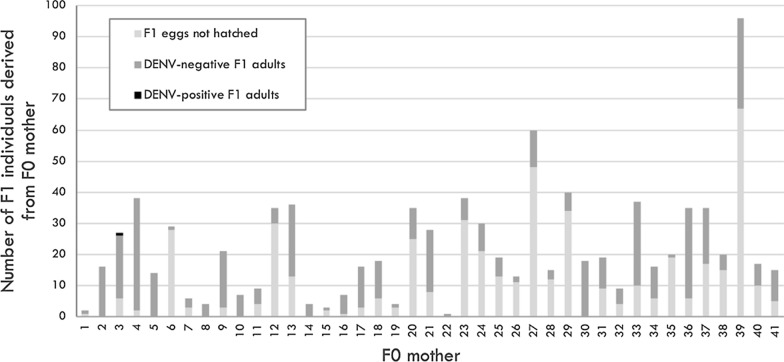

## Background

Dengue is a mosquito-borne viral disease. There are an estimated 390 million infections per year, and 3.9 billion people living in 128 countries at risk of infection [1, 2]. Dengue is caused by one of four dengue virus (DENV) serotypes, which are transmitted primarily by *Aedes aegypti* and *Ae. albopictus* mosquitoes [3]. In recent years, both species have expanded their geographical distribution and, predictions of various climate change scenarios suggest their distribution will increase further. Such scenarios are likely to trigger emergence and re-emergence of arboviruses such as dengue worldwide [4].

DENV is sustained naturally in a horizontal, human-mosquito transmission cycle. Transmission of virus between humans *via* the mosquito vector is a by-product of a female mosquito’s need for protein to develop the eggs [5]. Vertical transmission (VT) of virus from mother to progeny may be an alternative mechanism for the virus to maintain itself in circulation in nature [6, 7]. Should there be transient suboptimal conditions for horizontal transmission (HT), some level of vertical transmission could increase the probability of the pathogen persisting [8]. In the laboratory, vertical transmission can even be observed over consecutive generations in *Ae. aegypti* [9]. Mosquito rearing temperature [10, 11], the strain of mosquitoes and the strain of virus have been suggested to influence vertical transmission [12, 13]. Despite this, several studies suggest that the frequency of vertical DENV transmission in the field is negligible [14, 15] or uncertain [16, 17].

While it is impossible to determine whether a virus-infected female caught from the field acquired the virus horizontally or vertically, female infection frequencies in nature can range from < 1% up to 10% [18–20]. As neither immatures nor adult males feed on human blood, detection of virus in these individuals, by default, is indicative of vertical transmission from mother to offspring. In the field, estimates of vertical transmission in this group vary from 0% [17, 19] up to around 20% in pooled larval samples or based on the Ovitraps Index (number of ovitraps with positive eggs divided by the total number of ovitraps installed) [14, 15].

To our knowledge, no studies have investigated whether vertically infected females are capable of re-establishing an infection in the natural mosquito-human transmission cycle during their next feeding event. Failing to produce viral loads high enough to transmit DENV to a naïve human host, a vertically infected female may effectively end up a dead-end host.

The objectives of this study were to estimate the proportion of F_0_ females that transmitted virus to their progeny after viremic blood meals from acutely infected dengue patients and the proportion of vertically infected F_1_ females that had infectious saliva. We assessed these vertical transmission indices in two sets of experiments, one testing individual field-reared *Ae. aegypti* and *Ae. albopictus* and their individual F_1_ progeny, after feeding on the viremic blood of 25 acutely infected dengue patients. With an additional five patient-derived blood meals, we compared the prevalence of DENV infection in pools of F_1_ mosquitoes produced by field-reared *Ae. aegypti*, and those reared under laboratory conditions.

## Methods

### Patient cohorts and healthy volunteers

Forty patient participants were enrolled between November 2018 and September 2019, with written informed consent obtained by qualified staff from Hospital for Tropical Diseases (HTD). The patients were enrolled on Ward D (male adult dengue ward) of HTD, based on the following inclusion criteria: (i) ≥ 15 years of age; (ii) an inpatient at HTD with < 96 h fever at the time of screening; (iii) clinical signs and symptoms consistent with dengue; and (v) written informed consent. Exclusion criteria were: (i) patients who were unconscious or severely ill; and (ii) pregnant women. Only enrolled patients who were NS1 rapid test-positive had venous blood drawn for blood-feeding mosquitoes. An aliquot of each blood sample was taken for quantification of virus and virus serotyping (see Dengue virus diagnostics below), and the remaining was transported to the insectary for blood-feeding.

Healthy volunteers, who also provided written informed consent, provided venous blood samples for experimental feeding (see “Infectious and non-infectious blood meals” below) and for colony maintenance of laboratory-reared mosquitoes. The tympanic temperature of all volunteers was measured prior to blood draws to confirm the healthy volunteers were afebrile.

### Experimental overview

To assess the frequency of VT, we performed two sets of experiments. The first set, using 35 patient-derived blood meals, aimed to compared VT frequency between the two species of field-reared mosquitoes *Ae. aegypti* and *Ae. albopictus*, in individual progeny. The second set, using only 5 blood meals, examined whether VT frequencies in *Ae. aegypti* are influenced by rearing conditions (field-reared *vs* laboratory-reared), in a slightly modified protocol, with testing for the presence of virus in pooled progeny.

All experiments began with viremic blood meals administered to adult mosquitoes to first infect them with DENV; experimental groups were always exposed in parallel. A second healthy blood meal was later offered to promote egg laying in those same mosquitoes. The first set of experiments administered this non-infectious blood meal on Day 14; the second set administered the blood meal 4 days earlier, on Day 10. All F_1_ eggs were collected and hatched in batches, according to each F_0_ female, while the whole body of each mother (F_0_) was collected and tested for DENV infection. After F_1_ mosquito emergence, individuals were harvested for DENV screening (see “Hatching and harvesting F_1_ mosquitoes” below). Screening of mosquitoes in the second set of experiments was performed in pools of up to 8 progeny, due to the large number of F_1_ individuals obtained.

### Origin of mosquitoes

Field-reared (F_0_) immature *Ae. aegypti* and *Ae. albopictus* mosquitoes were collected on a weekly basis in households within District 8, Ho Chi Minh City (HCMC), Vietnam. Only fourth-instar larvae or pupae were included in the experiment, to maximise their development time under field conditions. Where colony mosquitoes were used, they were reared under laboratory conditions, and were of the same HCMC genetic background. Laboratory-reared mosquitoes were from generations 45 and 46, but were outcrossed with 10% wild-type males every second generation to avoid inbreeding depression, and to maintain genetic variation in the population [20].

### Mosquito rearing

After collection from the field, fourth-instar larvae and pupae were transported directly to the insectary at Oxford University Clinic Research Unit, District 5, HCMC, where they completed development in the same water in which they were collected (with no additional food provided). After emergence, the field-reared mosquitoes were maintained under controlled environmental conditions as follows: 28 °C; 65–85% relative humidity; and a 12:12 h light:dark cycle with sugar and water *ad libitum*. All mosquitoes were confirmed as either *Ae. aegypti* or *Ae. albopictus* visually, based on morphological characteristics. *Aedes aegypti* mosquitoes were further confirmed at a molecular level, using *Ae. aegypti-*specific primers targeting the *RPS17* gene, in a multiplex RT-PCR that also targeted DENV (see Dengue virus diagnostics below). After emergence, up to 5 females and 5 males were maintained in individual cups, to allow mating. For maintenance, mosquitoes were allowed access to water and sucrose solution *ad libitum* until one day before blood-feeding (irrespective of the source of blood), when water and sugar were then removed to starve the females. The same rearing conditions were used for the colonised mosquitoes in the second set of experiments.

### Infectious and non-infectious blood meals

Up to 20 F_0_ mosquitoes were maintained in cups (1:1 ratio of females:males) and offered the blood of NS1-positive dengue patients *via* artificial membrane feeders. Venous blood, drawn into an EDTA tube, was collected from patients for viral quantification, serotyping and to be offered to mosquitoes within 1 h of the blood draw. After 30 min of access to the viremic blood, females were sorted and only fully engorged were retained. All mosquitoes were kept in cups under controlled conditions, as described above, until the non-infectious blood meal was offered to the surviving females. Blood from healthy donors was drawn and provided to the F_0_ females on Day 14 or Day 10, according to the experiment set. Only fully engorged females were retained and placed into individual cups containing wet cotton balls for oviposition.

### Hatching and harvesting F_1_ mosquitoes

Five to 7 days after the second blood-feeding, eggs from each F_0_ female were hatched in cups and kept in incubators until mosquito emergence, for both sets of experiments. For the first experiment with *Ae. aegypti* and *Ae. albopictus*, the whole body of males from F_0_ DENV-positive females were collected for DENV screening 14 days after mosquito emergence. For F_1_ females, saliva was collected for inoculation into five naïve mosquitoes, as previously described [21], before their whole bodies were collected for DENV screening. Inoculated mosquitoes were then harvested 7 days post-injection and screened for DENV in pools, according to the saliva source. For the second set of experiments, the F_1_ progeny was collected on Day 7 after emergence and whole bodies were screened for DENV in pools of up to 8 individuals, with males and females tested separately. DENV detection was performed as described in the following section.

### Dengue virus diagnostics

DENV plasma viremia levels in patient blood samples were measured by a validated, quantitative serotype-specific RT-PCR assay [22]. Only fully engorged females that laid eggs after the non-infectious blood meal were screened for DENV infection. Briefly, mosquitoes were homogenised with a 2 mm glass bead in squash buffer (10 mM Tris, 1 mM EDTA, 50 mM NaCl [pH 8.2] and proteinase K), followed by an incubation period of 56 °C for 10 min, 98 °C for 15 min and a cool down step to 15 °C. Each sample was tested in a duplex RT-PCR, designed to amplify a conserved 3’-UTR region of all four DENV serotypes, and the *Rps17* gene from *Ae. aegypti* as an internal control, as previously described [23], using a LightCycler480 Instrument (Roche, Mannheim, Germany) with the following run conditions: 50 °C for 15 min, 95 °C for 2 min, followed by 45 amplification cycles of 95 °C for 15 s, 60 °C for 30 s and a final cooling step of 40 °C for 10 s.

### Calculations of vertical transmission

We estimated three related indices of vertical transmission, to help understand different contributions that each has to transmission dynamics. First, we calculated the number of F_0_ mothers who are capable of transmitting virus to any number of their progeny. Secondly, we calculated the proportion of their progeny, to whom they successfully transmit that virus. And finally, we calculated the proportion of infected progeny that are capable of expectorating virus in their saliva, a necessary element for re-introducing virus back into the horizontal transmission cycle.

**Fig. 1:**
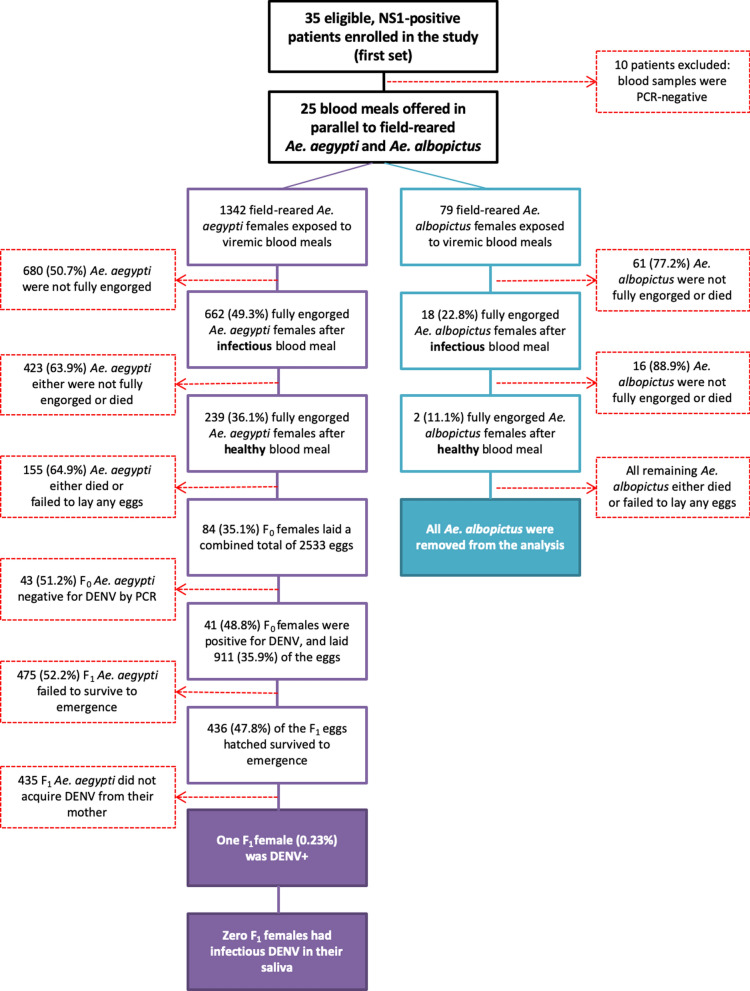
Flowchart of patient enrolment and mosquito processing to compare vertical transmission frequencies between field-reared *Ae. aegypti* and *Ae. albopictus* mosquitoes. The flowchart depicts the fate of mosquitoes as they were processed, in order to determine the frequency of vertical transmission of F_0_ females after feeding on blood from acutely-infected dengue patients admitted to the Hospital of Tropical Diseases (HTD) in Ho Chi Minh City, Vietnam. Boxes in red represent the samples excluded from analysis

We calculated these indices in two different ways, based on different denominators. The first formulas may be interpreted as providing a more refined estimate of vertical transmission, as it is based on a restricted subset of F_0_ mosquitoes with a confirmed DENV infection only, including all eggs derived from those females. The second, more inclusive estimate encapsulates all females who fed upon the infectious blood meal and laid eggs, irrespective of the eventual infectious status of the F_0_ female. This estimate is more conservative and is expected to more closely represent estimates derived from field-based studies. The precise formulas used to calculate these indices are listed below. The numbers in brackets at the end of the equation represents the number of mosquitoes used as the denominator in each equation. The bold text represents the equation (and denominator) used for the more inclusive, and thus conservative estimates of vertical transmission. All values can be found in the correspond flowchart (Fig. 1).

1 $$\begin{aligned} & {\mathbf{Frequency}}\, {\mathbf{of}}\,{\mathbf{mothers}}\,{\mathbf{that}}\,{\mathbf{transmit}}\,{\mathbf{virus}}\,{\mathbf{to}}\, {\mathbf{any}}\, {\mathbf{of}}\, {\mathbf{their}}\, {\mathbf{progeny}} \hfill \\ &= \frac{\text{Number of DENV infected mothers with at least one DENV positive progeny}}{{{\text{Total number of DENV infected }}\left( { + {\mathbf{uninfected}}} \right) {\text{mothers that laid eggs after second blood meal }}\left( {41/{\bf 84}} \right)}} \end{aligned}$$

2 $$\begin{aligned} & {\mathbf{Proportion}}\, {\mathbf{of}}\, {\mathbf{F}}1\, {\mathbf{progeny}}\, {\mathbf{that}}\, {\mathbf{inherited}}\, {\mathbf{virus}} \,{\mathbf{from}} \,{\mathbf{infected}}\, {\mathbf{mothers}} \hfill \\ &= \frac{{{\text{Number of surviving F}}1 {\text{progeny that were infected with virus}}}}{{{\text{Total number of eggs laid by all DENV infected }}\left( { + {\mathbf{uninfected}}} \right) {\text{mothers }}\left( {911/\bf2533} \right)}} \hfill \\ \end{aligned}$$

3 $$\begin{aligned} & {\mathbf{Proportion}}\, {\mathbf{of}}\, {\mathbf{infected}}\,{\mathbf{female}}\,{\mathbf{progeny}}\, {\mathbf{capable}}\, {\mathbf{of}} \,{\mathbf{transmitting}}\, {\mathbf{virus}}\, {\mathbf{horizontally}} \hfill \\ &= \frac{\text{Number of DENV infected female progeny with infectious DENV in their saliva}}{{{\text{Total number of eggs laid by all DENV infected }}\left( { + {\mathbf{uninfected}}} \right) {\text{mothers }}\left( {911/\bf2533} \right)}} \hfill \\ \end{aligned}$$

## Results

### Patient enrolment

We enrolled a total of 40 NS1-positive patients. RT-PCR was performed retrospectively to determine the viremia and virus serotype. Of the 30 patients’ blood samples in whom we could confirm virus infection by RT-PCR, the predominant serotype was DENV-2 (50%) with the range of viremia of 5.70–7.51 log_10_ copies/ml. The second most predominant serotype was DENV-1 (30%) with viremia of 5.31–7.97 log_10_ copies/ml and lastly was DENV-4 (20%) with viremia of 6.67–8.32 log_10_ copies/ml. Characteristics of the enrolled patients are found in Additional file 1: Table S1. Ten patient blood samples were excluded from the study because they were DENV-negative when tested by RT-PCR.

### Species composition of field-reared mosquitoes

Between November 2018 and September 2019, we collected a total of 3720 *Aedes* larvae/pupae over a period of 38 weeks, from up to 4 collection sites in District 8, HCMC, each week. The predominant species was *Ae. aegypti* with 3528 (94.8% of total) individuals collected (1802 females and 1726 males). On average, 47 *Ae. aegypti* female mosquitoes were available for experiments on a weekly basis. We also collected 192 *Ae. albopictus* mosquitoes (5.16% of total), with 79 females and 114 males. The majority of remaining individuals collected were culicine mosquitoes.

### Estimates of vertical transmission in field-reared *Ae. aegypti* and *Ae. albopictus*

We examined vertical DENV transmission by field-reared *Ae. aegypti* and *Ae. albopictus* after they had fed on viremic blood meals. The 25 independent viremic blood meals resulted in 662 of 1342 F_0_ *Ae. aegypti* females (49.3% of the total) being engorged. Approximately one third of these females (*n* = 239) fed upon the non-infectious blood meal, administered on Day 14, which stimulated the second gonotrophic cycle. Of these 239 F_0_ females, 84 survived long enough to lay a total of 2533 eggs. Retrospectively, we determined that only 41 of these 84 females were infected with DENV. The eggs (*n* = 911) from these 41 DENV infected F_0_’s developed into 436 mature adults. All 214 F_1_ males were negative for DENV infection. Only one (CI: 0.00–0.03) of the 222 F_1_ females tested positive for DENV in her body; the latter was infected with DENV-2 serotype and had a Cq value of 33.11. None of the saliva samples collected from any F_1_ female contained infectious virus that amplified in the *in vitro* transmission assay (Fig. 1).

**Fig. 2:**
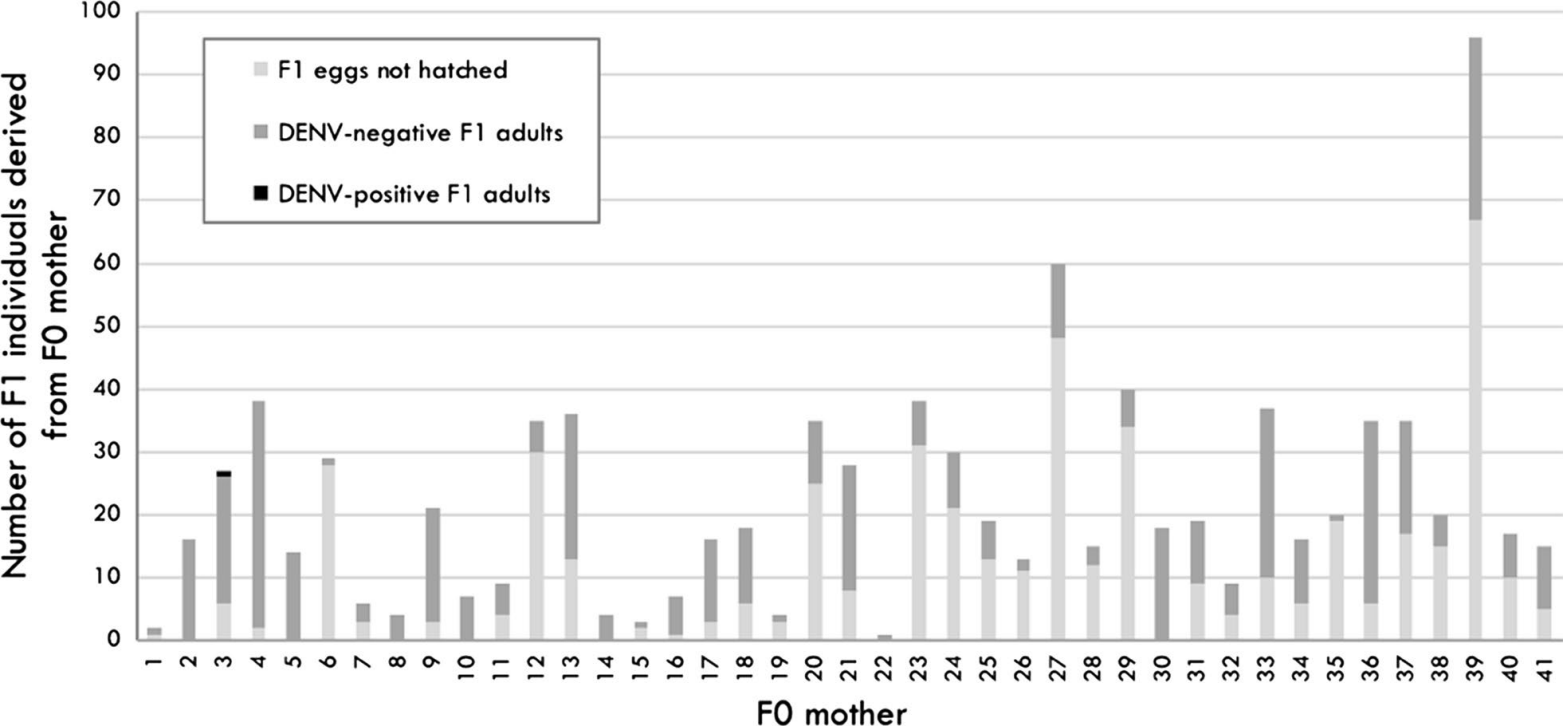
Histogram showing the number of F_1_ derived from each F_0_ female, field-reared and DENV-infected, *Ae. aegypti*. Each bar represents the progeny of each of the 41 F_0_ females DENV-positive, separated by eggs no hatched (light-grey), F_1_ DENV-negative (dark-grey) and F_1_ DENV-positive (black)

Thus, our results suggest that vertical transmission under conditions similar to the natural history is rare; for every 41 DENV-infected, field-reared females that survive and reproduce after 14 days, only 1 (2.4%) would transmit virus to the progeny. If we calculate based on the F_1_ progeny that emerged to adults (*n* = 436), the percentage of DENV-infected progeny from a DENV-infected mother drops to 0.23% (Fig. 1). Using a more conservative denominator, which includes all females that fed upon an infectious blood meal and laid eggs, regardless their infection status, this represents 1 female who transmits DENV to her progeny for every 84 females (1.2%). With only 1 of all the 911 eggs laid by an infected mother having acquired virus, we calculate that 0.11% of progeny born to a DENV-infected mother in the second gonotrophic cycle would carry DENV (Fig. 2).

For *Ae. albopictus*, 18 of 79 females (23.0%) who fed on the infectious blood meal survived and fed upon the second, healthy blood meal. However, only 2 of the 18 females (11.1%) become engorged, and neither produced a second gonotrophic cycle. As such, we were unable to assess the frequency of vertical transmission of DENV in *Ae. albopictus* (Fig. 1).

### Vertical transmission frequencies of field-reared *vs* laboratory-reared *Ae. aegypti* females

In this second set of experiments, we exposed an additional 460 field-reared *Ae. aegypti* females, alongside 946 laboratory-reared *Ae. aegypti* females, to 5 patient-derived blood meals. We tested pools of female progeny, rather than individual mosquitoes, for the presence of DENV infection.

At Day 10 post-virus exposure, the surviving 222 engorged field-reared females were exposed to healthy blood meals, of which 67 females (30.2%) fed to repletion. Eighteen females were positive for DENV and laid 879 F_1_ eggs in total. A total of 558 F_1_ mosquitoes were tested in 108 pools for DENV infection and all tested negative. For laboratory-reared females, there were 571 engorged individuals after the first infectious blood meal and 205 after the second one. In total, 72 females were both infected with DENV and laid eggs, from which 3240 mature F_1_ mosquitoes were reared. All 564 pools for screening were again test-negative for DENV (Additional file 2: Figure S1).

## Discussion

Our data show that less than 3% of field-reared females who are known to have fed upon a patient-derived viremic blood meal, are capable of transmitting DENV to any of their progeny two weeks later. We further demonstrate that fewer than one quarter of a percent of mosquitoes born to these virus-exposed mothers will acquire infection. If we calculate the frequency using all eggs laid by females both infected and uninfected that were exposed to the same blood meals, this number goes to less than 1 of 2500 eggs laid. Finally, even less are likely to re-introduce virus into the human population.

The role of vertical virus transmission has been considered under multiple scenarios. It has been stimulated by theory that transmission of virus through the germ line may provide an alternate mechanism for virus maintenance in nature. Some studies suggest that vertical transmission allows re-emergence and maintenance of arboviruses in a vector population in different scenarios: (i) in between non-epidemic periods; (ii) when the density of mosquito population is low (due to winter diapause); or (iii) in the absence of any viremic hosts [24, 25]. It has also been suggested that virus in diapausing eggs can remain viable until mosquito emergence, thus the virus can multiply and be re-introduced horizontally to the vertebrate host population [26, 27].

While vertical virus transmission in the field has been documented since it was first described in 1906 [28–30], there is insufficient evidence to demonstrate it contributes to re-emergence or maintenance of viruses in nature. A recent study described DENV vertical transmission in field-collected immature mosquitoes by detecting the virus in 0.4% of the pooled samples, and even this low VT frequency could explain the persistence of the virus during interepidemic times in Africa [31]. However, those studies have failed to test the important hypothesis, that vertically acquired virus can actually be horizontally transmitted [8].

Mimicking natural transmission dynamics as closely as possible and using field-reared mosquitoes exposed to blood drawn from acutely viremic dengue patients, our data support the premise that vertical transmission is a relatively rare occurrence [8, 31, 32]. Moreover, we could not detect virus in the saliva of our single, vertically-infected female. Our mosquito sample size was relatively small, but based on our data, it suggests that re-introduction of the virus to the human population *via* horizontal transmission is an uncommon event. Alternative mechanisms to explain re-emergence of virus within a human population may be introduction of virus from another location, or continued asymptomatic carriage in the human population.

A stable and systemic dissemination of viruses acquired by oral feeding in *Aedes* mosquitoes is important as a determinant for VT to subsequent progeny [33, 34]. This dissemination can take days or weeks to occur [35, 36] and thus most vertical transmission must occur in the female later gonotrophic cycles [32, 33, 37]. Before stimulating a second gonotrophic cycle with a healthy blood meal, we allowed 14 days for virus replication, a period sufficiently long to achieve systemic viral dissemination, and high viral titres, in our infection model [21, 38, 39]. Despite this, the proportion of DENV-infected progeny we detected was still below 0.5%. Future experiments should consider stimulation of more frequent and additional gonotrophic cycles, to promote cell proliferation and associated viral replication. This may assist detection of an increased prevalence of vertical transmission of virus from a DENV-infected mother to her progeny.

Previous studies have reported notably high prevalence of vertical transmission and suggest a significant contribution of this phenomenon to the maintenance of DENV in nature. One recently published estimate of VT ranges from 14.8 to 23.5% in *Ae. aegypti* collected in ovitraps [14]. Yet these values are derived from calculations of the number of positive ovitraps, rather than the number of positive individuals contained within that trap. With an experimental design more similar to our own study, Sánchez-Vargas et al. [34] reported virus infection frequencies varying from 55 to 68.6% in F_1_ mosquitoes derived from infected mothers. They did however use a blood meal spiked with DENV-2 to first infect mosquitoes, and tested F_1_ mosquitoes in pools, rather than individually [34]. Pooling of samples can lead to an overestimation of the frequency of occurrence of VT and reduces the sensitivity of DENV-detection in those samples [8].

A limiting factor in achieving a large sample size in this study was our restricted criteria for ‘field-reared’ mosquitoes by selecting only fourth-instars and larvae, as we intended to maximise the duration of their development under field conditions. We also observed relatively high mortality after the second blood meal, prior to F_0_ females laying eggs, for the field-reared mosquitoes. One possible reason for this is the females’ age, in combination with variable and suboptimal rearing conditions during the larval stage in the field. Such suboptimal conditions may include nutritional stress, over-/under-crowding and fluctuating temperatures which can influence fitness and longevity [39–41], further highlighting that vertical transmission in the field is likely an uncommon event. As such, while our sample sizes are small, we are confident that the experimental design and resulting data are representative of realistic conditions.

## Conclusions

To the best of our knowledge, this is the first study to assess vertical transmission of DENV using field-reared *Ae. aegypti* mosquitoes exposed to viremic blood from patients. Our data demonstrate infrequent transmission of virus from mother to progeny and can suggest that vertically acquired virus may not play a substantial role in dengue virus transmission vertically.

## Supplementary information

**Additional file 1: Table S1.** Characteristics of the 40 dengue patients enrolled in the study.

**Additional file 2: Figure S1.** Flowchart of patient enrolment and mosquito processing to compare vertical transmission frequencies between field- and laboratory-reared *Ae. aegypti* mosquitoes. The flowchart depicts the fate of mosquitoes as they were processed, in order to determine the frequency of vertical transmission of F_0_ females after feeding on blood from acutely-infected dengue patients admitted to the Hospital of Tropical Diseases (HTD) in Ho Chi Minh City, Vietnam. Boxes in red represent the samples excluded from analysis.

## Data Availability

All relevant data generated or analysed during this study are included in this published article and its additional files.

## Abbreviations

VT: vertical transmission
HT: horizontal transmission
HTD: Hospital for Tropical Diseases
EC: Ethics Committee
NS1: non-structural protein 1
HCMC: Ho Chi Minh City
RT-PCR: reverse transcriptase polymerase chain reaction
3’UTR: 3 prime untranslated region
