# Supplementary material for: Assessing the vertical transmission potential of dengue virus in field-reared Aedes aegypti using patient-derived blood meals in Ho Chi Minh City, Vietnam
Source: Parasit Vectors. 2020 Sep 14;13:468. doi: 10.1186/s13071-020-04334-5 (PMC7490885; doi:10.1186/s13071-020-04334-5)
Supplement: Supplementary file 1 — Additional file 1: Table S1. Characteristics of the 40 dengue patients enrolled in the study. [file 13071_2020_4334_MOESM1_ESM.docx]

**Additional file 1: Table S1.** Characteristics of the 40 dengue patients enrolled in the study

| **Characteristics** | **Undefined** | **DENV-1** | **DENV-2** | **DENV-4** | **All patients** |
| --- | --- | --- | --- | --- | --- |
|  | **(*n* = 10)** | **(*n* = 9)** | **(*n* = 15)** | **(*n* = 6)** | **(*n* = 40)** |
| Age (years) | 28.0 (25.5–34.7) | 33.5 (25.2–35.0) | 34.0 (27.0–39.0) | 36.5 (27.7–37.7) | 34.5 (26.0–38.0) |
| Day of illness  2  3  4 | 0  3 (30.0)  7 (70.0) | 0  3 (33.3)  6 (66.6) | 4 (26.7)  2 (13.3)  9 (60.0) | 0  3 (50.0)  3 (50.0) | 4.0 (10.0)  11.0 (27.5)  25.0 (62.5) |
| Viremia (log_10_ copies/ml) | < LOD | 7.5 (5.3–8.0) | 6.9 (5.7–7.5) | 7.6 (6.7–8.3) | 7.2 (6.3–8.1) |

*Notes*: For age and viral load, values are presented as median (interquartile range). Day of illness are presented as number of patients (percent of total patients within that column). LOD: limit of detection. LOD for DENV-1 = 5 copies per reaction; DENV-2 = 1 copy per reaction; DENV-3 = 5 copies per reaction; DENV-4 = 10 copies per reaction [22].
