# Supplementary figures and images for: Assessing the vertical transmission potential of dengue virus in field-reared Aedes aegypti using patient-derived blood meals in Ho Chi Minh City, Vietnam
Source: Parasit Vectors. 2020 Sep 14;13:468. doi: 10.1186/s13071-020-04334-5 (PMC7490885; doi:10.1186/s13071-020-04334-5)

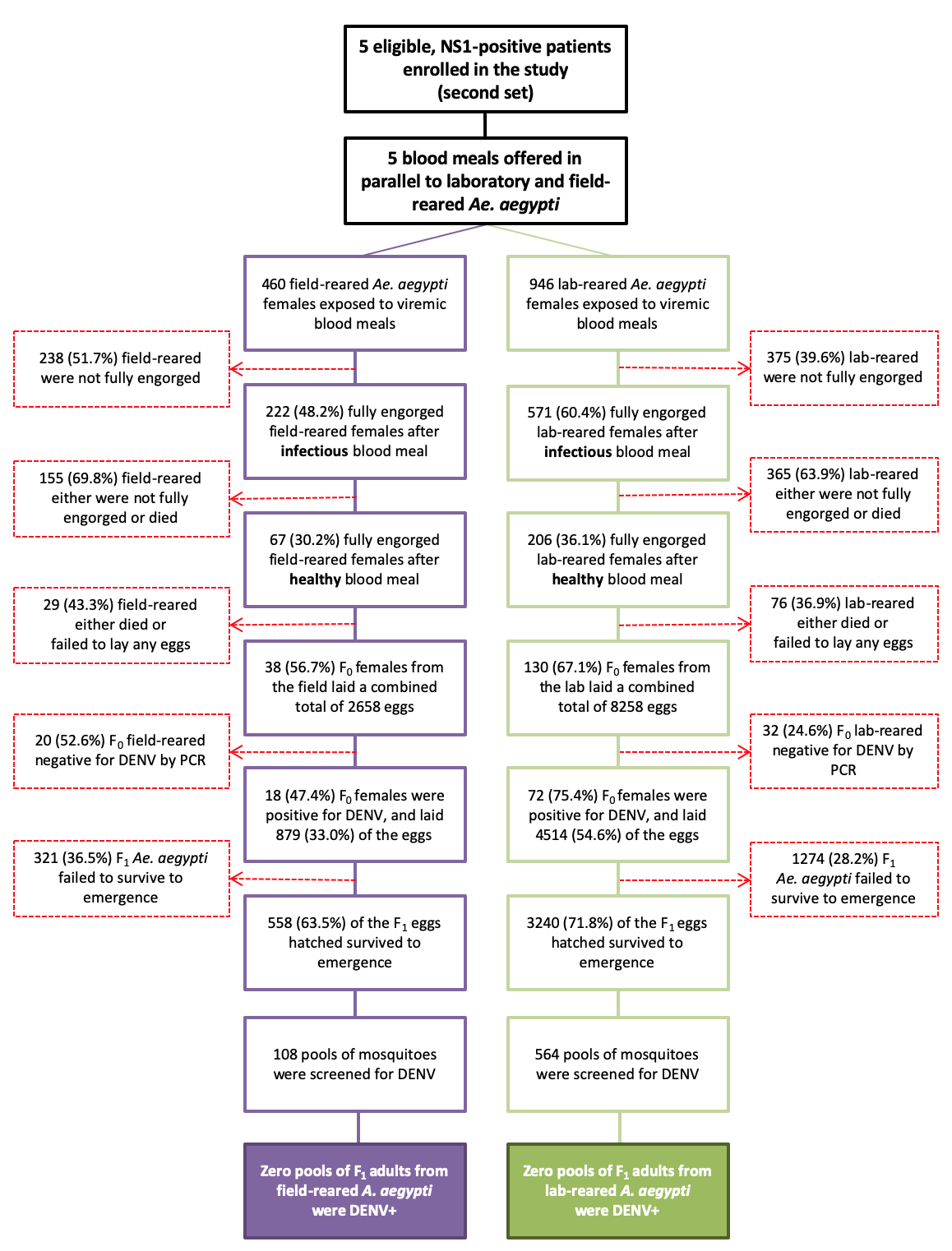

Supplement: Supplementary file 2 — Additional file 2: Figure S1. Flowchart of patient enrolment and mosquito processing to compare vertical transmission frequencies between field- and laboratory-reared Ae. aegypti mosquitoes. The flowchart depicts the fate of mosquitoes as they were processed, in order to determine the frequency of vertical transmission of F0 females after feeding on blood from acutely-infected dengue patients admitted to the Hospital of Tropical Diseases (HTD) in Ho Chi Minh City, Vietnam. Boxes in red represent the samples excluded from analysis. [file 13071_2020_4334_MOESM2_ESM.png]
